# Regulatory mechanisms of phosphatase of regenerating liver (PRL)-3

**DOI:** 10.1042/BST20160146

**Published:** 2016-10-19

**Authors:** Teresa Rubio, Maja Köhn

**Affiliations:** Genome Biology Unit, European Molecular Biology Laboratory, Meyerhofstrasse 1, 69117 Heidelberg, Germany

**Keywords:** cancer, dual-specificity phosphatases, metastasis, post-translational modification, protein phosphatases, PTP4A3

## Abstract

The phosphatase of regenerating liver (PRL)-3 is overexpressed in many human cancer types and tumor metastases when compared with healthy tissues. Different pathways and mechanisms have been suggested to modulate PRL-3 expression levels and activity, giving some valuable insights but still leaving an incomplete picture. Investigating these mechanisms could provide new targets for therapeutic drug development. Here, we present an updated overview and summarize recent findings concerning the different PRL-3 expression regulatory mechanisms and posttranslational modifications suggested to modulate the activity, localization, or stability of this phosphatase.

## Introduction

The phosphatase of regenerating liver (PRL)-3 protein belongs to the PRL subfamily, which are dual-specificity phosphatases classified as a subgroup of protein tyrosine phosphatases (PTP). This subfamily of small proteins (∼22 kDa) includes three members, PRL-1, -2, and -3. Rat PRL-1 was the first member described in 1994 as a gene induced in mitogen-stimulated cells and regenerating liver [1]. This discovery led to the identification of PRL-2 and PRL-3, exhibiting 87 and 76% of sequence identity when compared with PRL-1 [2]. All PRLs promote cell proliferation, migration, invasion, tumor growth, and metastasis, and have been proposed as potential biomarkers of cancer progression [3]. As the first of the three PRLs, PRL-3 was found overexpressed in liver metastasis from colon cancer but not in normal colon tissue or in primary tumors [4]. This finding has drawn much attention to this protein, and PRL-3 is the most studied of the three PRLs at this point [3].

In addition to colon cancer, PRL-3 was also found to be overexpressed in many other human cancer types, such as Hodgkin's lymphoma [5], melanomas [6], acute myeloid leukemia (AML) [7], gastric [8], ovarian [9], breast [10], and esophageal squamous cell carcinoma [11]. In recent years, an increasing number of reports have implicated PRL-3 as an activator of pathways involved in proliferation, invasion, and cell motility, such as phosphatidylinositol-3 kinase (PI3K)/serine threonine protein kinase AKT [12], the tyrosine protein kinase Src [13], Rho GTPases family [10], mitogen-activated protein kinase/extracellular signal-regulated kinase (MAPK/ERK) kinase (MEK) [14], and epidermal growth factor receptor (EGFR) signaling [15], as well as mediating epithelial mesenchymal transition (EMT) by down-regulating phosphatase and tensin homolog (PTEN) [16] or the cadherin family [17,18]. Interestingly, PRL-3 can also recruit endothelial cells participating in tumor angiogenesis, an essential event for cancer progression [19]. In spite of these insights, the exact molecular mechanisms by which PRL-3 affects these pathways remain unclear. However, it is well established that the enzymatic activity of PRL-3 is essential for its function since the inactive mutant (C104S) neither activates these pathways nor promotes cancer metastasis [20].

Taken together, these studies demonstrate that PRL-3 overexpression is a key contributor to cancer progression, invasion, and metastasis, but the mechanisms underlying the regulation of PRL-3 expression in physiological and pathological conditions still need to be determined. Elegant studies described the amplification of the PRL-3 gene (*PTP4A3*) in the chromosomal region 8q24.3 in colon cancer metastases [4], breast [21], and gastric carcinomas [22]. Gene amplification is a mechanism to enhance gene expression of oncogenes and could explain PRL-3 overexpression in some human cancer types [4]. Nevertheless, exon sequencing of 10 samples from AML with high levels of PRL-3 did show neither gene amplification nor somatic mutations in PRL-3 [23], suggesting that transcriptional, translational, or posttranslational regulation might be involved in the aberrant expression of PRL-3.

**Figure 1.**
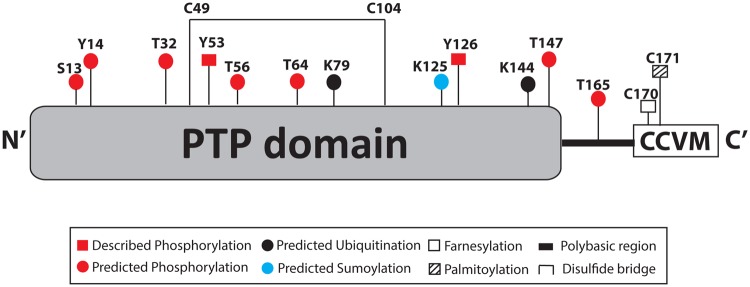
Schematic representation of the posttranslational modifications described or predicted for PRL-3.

Here, we give an updated overview on the regulation of PRL-3 focusing on mechanisms of posttranslational regulation. Table 1 gives an overview of the different levels and molecules involved in PRL-3 regulation, and Figure 1 shows the posttranslational modifications described or predicted for PRL-3.

**Table 1:** Summary of the different molecules involved in PRL-3 regulation

Type	Regulator	Function	References
Transcriptional	P53	Enhancer	[24]
	TGFβ	Down-regulator	[12]
	Snail	Enhancer	[26]
	VEGF	Enhancer	[27]
	STAT3	Enhancer	[23]
	miRNA-495	Enhancer	[30]
Translational	PCBP1	Down-regulator	[31]
Posttranslational	TRP32	Activator	[34]
	Src	Activator	[35]
	PKC	Predicted phosphorylation	[2]
	CK2	Predicted phosphorylation	[2]
	Farnesyltransferase	Plasma membrane localization	[38]
	Palmitoyltransferase	Plasma membrane localization	[41]
	FKBP38	Proteasome degradation	[43]
	USP4	Prevention of degradation	[46]

## PRL-3 transcriptional regulators

The first PRL-3 transcriptional regulator reported was the tumor suppressor p53 as an activator of PRL-3 transcription [24]. Interestingly, in the present study, it was also found that PRL-3 is both a positive and negative regulator of the cell cycle in a dose-dependent manner, highlighting the importance of fine tuning the regulation of PRL-3 protein levels. However, it was determined that the incidence of PRL-3 in some cancer types, such as AML, is higher (50%) than that of p53 mutations (10–15%) [25], suggesting that other regulators could also control PRL-3 expression. Confirming this hypothesis, transforming growth factor β (TGFβ) was identified as an inhibitor of PRL-3 transcription, by mediating the binding of the transcription factors Smad2 and Smad3 to the PRL-3 promoter in cancer cells. In this proposed model, TGFβ acts as a tumor suppressor, and its loss would lead to PRL-3 overexpression and cancer progression [12].

Moreover, Snail, a transcription factor member of the C2H2-type zinc finger family, is able to bind to the PRL-3 promoter enhancing its transcription and subsequently its protein levels in human colon cancer cells [26]. Interestingly, it was reported previously that PRL-3 overexpression also enhances Snail expression in colon cancer cell lines [18], suggesting that PRL-3 could participate in a positive feedback loop with Snail.

Previously, a novel model was proposed in which cancer cells expressing high levels of PRL-3 were associated with the release of high levels of vascular endothelial growth factor (VEGF), leading to angiogenesis [27]. VEGF induces myocyte enhancer transcription factor 2 (MEF2C) expression, which binds to the PRL-3 promoter enhancing its transcription. This model also involved PRL-3 in angiogenesis-related processes.

Most recently, it was reported that the signal transducer activator of transcription 3 (STAT3) [23] positively regulates PRL-3 transcription and expression in leukemia cells. Previous reports also showed that the PRL-3 protein contributes to STAT3 activation [13,28,29], suggesting again that PRL-3, as described above for Snail, could participate in a positive autoregulatory feedback loop.

In addition to transcription factors, the involvement of miRNAs in the transcriptional regulation of PRL-3 was recently described. Li et al. [30] discovered that miRNA-495 down-regulates PRL-3 mRNA and protein levels in gastric cancer cells.

## PRL-3 translational regulation

It was observed that PRL-3 protein levels in colon, breast, lung, and other cancer types do not correlate well with its mRNA levels, suggesting that PRL-3 is not only regulated at the transcriptional level [31]. In this regard, it was shown that poly (C)-binding protein 1 (PCBP1), a member of the hnRNP family of RNA- and DNA-binding proteins, interacts with the 5′-UTR region of PRL-3 mRNA, down-regulating its translation [31]. Interestingly, PCBP1 cannot completely inhibit PRL-3 translation, suggesting existence of other negative regulatory mechanisms when PRL-3 protein is absent, but its mRNA is present [31].

## PRL-3 posttranslational modifications and regulators

Several new insights in posttranslational regulation have emerged in recent years, increasing the complexity of PRL-3 regulation. Posttranslational modifications can modulate protein stability as well as affect its subcellular distribution, activity, or interactions with other proteins.

### The regulation of PRL-3 activity by the addition or modification of small chemical groups

#### Oxidation and reduction in the PRL-3 catalytic cysteine

The PRL proteins undergo oxidation and inactivation as shown first for the PRL-1 catalytic domain [32], a property which is also displayed by many other PTPs [33]. PRL-3 can form a reversible disulfide bridge between the catalytic cysteine (C) Cys104 and the proximal Cys49 under oxidative conditions. Within cells, oxidized proteins are reduced by thioredoxin (TRX) or TRX-related proteins using electrons donated by the glutathione and TRX reductases (TrxR), which use the electrons supplied by the NADH. In this context, TRP32 (a TRX-related protein) reduces the PRLs protecting them from oxidation and, in consequence, maintaining their activity and thus contributing to cancer progression and metastasis [34].

#### PRL-3 phosphorylation

Previously, PRL-3 was found to be phosphorylated in colon cancer cells [35]. This phosphorylation is carried out by the *sarcoma* (Src) proto-oncogene, a tyrosine protein kinase, as was demonstrated using Src inhibition, Src/Yes/Fyn-deficient (SYF) mouse embryo fibroblasts [36] and overexpressing Src in the latter. The overexpression of different PRL-3 mutants, in which each of the six predicted phosphotyrosine (Y) residues were mutated to nonphosphorylatable phenylalanine (F) residues, led to the identification of Tyr53, a site previously predicted by Zeng et al. [2] as a Src kinase target. Moreover, the Src-mediated phosphorylation of Tyr53 is essential for PRL-3 invasion and motility in SW480 colon cancer cells, since Y53F mutant overexpression or Src inhibitor treatment suppressed invasion and motility when compared with cells overexpressing *wt* PRL-3 or untreated cells. Interestingly, as Tyr53 is present in all PRLs and is surrounded by a highly conserved sequence, this provides a new research direction for studies of the regulation of PRLs by phosphorylation. The biochemical mechanisms and structural consequences of PRL-3 phosphorylation still need to be investigated. The authors hypothesized that Tyr53 phosphorylation could change the affinity of PRL-3 for a specific site in the plasma membrane. This modification could also modify its catalytic activity or stimulate the interaction with other proteins, suggesting that PRL-3 could also function as a scaffold protein. Moreover, Tyr53 is near the active site of PRL-3 and was previously suggested to participate in substrate-binding in a phosphorylation-dependent manner [37]. In addition to Tyr53, Fiordalisi et al. also found Tyr126 phosphorylated in PRL-3. However, the kinase involved and the role of this phosphorylation remain unknown [35]. Interestingly, PRL-3 activates Src by inhibiting its regulatory protein tyrosine kinase, C-terminal Src kinase [13]. Taken together, this suggests that PRL-3 could participate in a positive feedback loop for Src activation.

In addition, other phosphorylated residues in mouse PRL-3 were predicted in 1998 using the ScanProsite software [2]. Two potential sites predicted to be phosphorylated by protein kinase C (Ser13 and Thr165) and four sites predicted for casein kinase II-dependent phosphorylation (Thr32, Thr56, Thr64, and Thr147) [2]. Finally, in PhosphoSite Plus (www.phosphosite.org), Tyr14 was also predicted as a phosphorylated residue in PRL-3.

Taken together, these observations suggest that phosphorylation may play an important role in PRL-3 regulation. However, further studies must be carried out in order to corroborate the predicted phosphorylation sites, as well as to define the role(s) of each phosphorylation in PRL-3 regulation.

### Regulation of PRL-3 localization by the addition of a hydrophobic chemical functional group

Prenylation of the PRLs was predicted in 1998 when the authors found the prenylation consensus motif CaaX in the C-terminus, where C is cysteine, a is an aliphatic amino acid, and X is any amino acid [2]. The canonical pathway is derived in three steps: (1) prenylation on the cysteine (C), (2) proteolysis of the aaX peptide, and (3) subsequent carboxymethylation of the C-terminus [38].

Two types of prenylation modifications exist: farnesylation, carried out by the farnesyltransferase enzyme (FT), and geranylgeranylation, catalyzed by geranygeranyl transferases (GGT I or GGT II). PRL-1 and PRL-2 can be farnesylated and geranylgeranylated [39]. PRL-3 is also farnesylated, but data on geranylgeranylation are contradictory [39,40]. PRL-3 farnesylation localizes the protein in the plasma membrane, early endosomes [39], and also to the Golgi [41], and the use of an FT inhibitor leads to PRL-3 redistribution to the nucleus. This is due to the presence of a polybasic sequence that could act as nuclear localization signal (NLS) close to the C-terminus. Thus, farnesylation may mask the NLS, retaining the proteins bound to membranes. These findings suggest that PRL-3 may have a role in the plasma membrane, Golgi, and early endosomes.

It was also reported that farnesylation of the CaaX motif may contribute to PRL-3 oligomerization mediating interaction through hydrophobic forces [42]. The authors speculated that this oligomerization of PRL-3 could limit the access of the substrate and, in consequence, decrease PRL-3 activity [42]. It was shown that PRL-1 crystallizes in trimers [43,44] and that it oligomerises in cells in a C-terminal farnesylation-dependent manner, which is necessary for its function [45]. On the other hand, PRL-3 NMR structures show a monomeric protein [37,46], but it was also shown to oligomerise in cells [42,45]. Nevertheless, more experiments have to be done to understand the role of oligomerization in modulating PRL-3 activity, as well as to study the protein–protein interactions that could also be involved in PRL-3 regulation.

In addition to farnesylation, the high homology of the PRLs with the cell division control 42 (Cdc42) protein, a member of the Rho GTPase family [2], has led to the proposal that PRL-3 is palmitoylated in the second cysteine of the CaaX motif (CCVM), and that it could bypass the post-prenylation steps of proteolysis and carboxymethlation [47]. Palmitoylation is the covalent attachment of palmitic acid to a cysteine residue of prenylated proteins. In contrast with prenylation, this modification is reversible altering the subcellular localization of the protein, regulating intracellular trafficking and/or protein–protein interactions [48]. Interestingly, the prenyl and palmitoyl modifications found in bCdc42 (brain isoform of Cdc42) and PRL-3 are not conserved in all proteins with a CCaX motif, indicating that this tandem modification is selective and may play a role in the regulation of these proteins [47]. The palmitoylation of bCdc42 enriches it in the plasma membrane enhancing its activity. However, more studies have to be done to understand the role of PRL-3 palmitoylation and to answer if it could enhance PRL-3 activity in the plasma membrane. Potentially, it would open the opportunity to investigate new PRL-3 palmitoylation inhibitors as cancer therapeutic drugs.

### PRL-3 stability regulation by covalent addition of a peptide (ubiquitination)

The first evidence of PRL-3 protein stability modulation was the discovery of peptidyl prolyl *cis*/*trans* isomerase (PPIase) FK506-binding protein 38 (FKBP38) as a mediator of PRL-3 degradation by the proteasome [49]. PRL-3 interacts with the N-terminal region of FKBP38. The PPIase domain resides in this region, but since FKBP38 does not have PPIase activity, it was suggested to act as a scaffold to mediate the degradation of PRL-3 by other unknown proteins [49].

Furthermore, other studies suggest that PRL-3 participates in an autophagy feedback mechanism, where accumulation of PRL-3 activates canonical autophagy and consequently becomes degraded by it [50]. Moreover, levels of PRL-3 correlated with high levels of autophagy-related genes in ovarian cancer tissue samples and with higher pathological stage, suggesting that autophagy plays a role in PRL-3-mediated ovarian cancer progression [50]. These insights suggest a complex regulation of PRL-3 protein levels by different degradation pathways, as well as PRL-3's participation in many molecular mechanisms within the cell in order to facilitate cancer progression.

Nevertheless, none of these studies suggest a posttranslational modification that is involved in targeting PRL-3 for proteasome or autophagy degradation, or explain the stimulus that leads to its degradation. Ubiquitination tags proteins for both proteasomal and autophagic degradation, suggesting that PRL-3 could be a target for protein ubiquitination. In this regard, the BDM-PUB ubiquitination site prediction 1.0 software (http://bdmpub.biocuckoo.org/) predicts four potential ubiquitinated lysines (K) within the human PRL-3 amino acid sequence, and two of them with more than 1.5 score of prediction: Lys79 and Lys144.

Confirming this prediction, the co-expression of PRL-3-expressing plasmids with HA-tag ubiquitin led to the detection of polyubiquitinated PRL-3 products [51]. Moreover, MG132 treatment, a proteasome inhibitor, increased the levels of PRL-3 confirming the previous findings of PRL-3 degradation by the proteasome pathway [49]. The authors also showed that ubiquitin-specific protease 4 (USP4) interacts with PRL-3 and decreases the levels of its ubiquitinated product preventing PRL-3 degradation in human cancer cells. Accordingly, USP4 and PRL-3 protein levels positively correlate in clinical samples and cancer cell lines, but not in the mRNA levels, confirming that the USP4-dependent regulation of PRL-3 protein levels is a posttranslational event [51].

These studies confirm that PRL-3 is degraded by the proteasome and/or the autophagy pathway, as well as that PRL-3 is a potential ubiquitin target protein. However, more studies are required to understand the molecular mechanism and stimuli behind this posttranslational modification, and which residues in PRL-3 are ubiquitinated. A protein can be monoubiquitinated, multiubiquitinated, or polyubiquitinated, each one targeting the protein for different processes. The polymerization of ubiquitin (poly-ubiquitination) can occur in residue Lys48 of ubiquitin targeting the protein for proteasome degradation, or in Lys67 targeting proteins for autophagy degradation [52,53]. The study of the molecular process of PRL-3 ubiquitination would help to better understand the mechanisms involved in PRL-3 protein degradation. Moreover, other ubiquitin-related modifications like the addition of small ubiquitin-like modifier (SUMO) peptide to a lysine residue of a target protein (SUMOylation) also modulate protein stability directly targeting proteins for degradation or facilitating or preventing its ubiquitination. In this regard, one SUMOylation consensus peptide (around Lys125) and one SUMO interaction site are predicted in the amino acid sequence of human PRL-3 using the GPS-SUMO prediction SUMOylation sites and SUMO-binding motifs 2.0 software (http://sumosp.biocuckoo.org/showResult.php).

In addition to protein stability, ubiquitination and SUMOylation also regulate protein–protein interactions, subcellular localization, and activity. Since a consensus lysine has been found in the amino acid sequence of PRL-3, as well as evidence for its ubiquitination, studying these modifications on PRL-3 would be of interest.

## Conclusions

**Figure 2.**
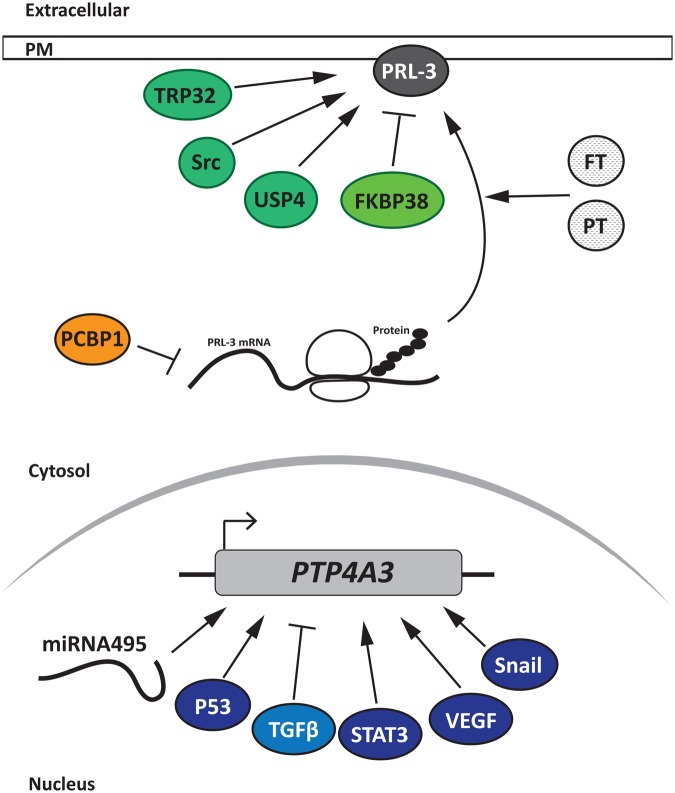
Representation of the different regulators of PRL-3 at transcriptional, translational, and posttranslational levels.

Knowing how PRL-3 is regulated is important to better understand how it participates in cancer progression. As we reviewed here, PRL-3 protein expression, localization, and activity are regulated by transcriptional, translational, and posttranslational mechanisms, which are represented in Figure 2. All these regulatory pathways could be co-ordinated in order to ensure the fidelity of PRL-3 expression within the cells. The alteration of any of these mechanisms might contribute towards increased PRL-3 expression and, in consequence, to cancer progression. In this regard, more studies must be carried out in order to improve our understanding of the mechanisms by which PRL-3 is regulated. Such studies could lead to the opening of a new therapeutic drug development field targeting these processes. Since PRL-1 and PRL-2, which also promote cancer progression, are much less studied in this regard, it would be interesting to investigate if their regulation involves similar mechanisms.

## Abbreviations

AKT = PKB, protein kinase B; AML, acute myeloid leukemia; bCdc42, brain isoform of the cell division control 42 protein; FKBP38, FK506-binding protein 38; FT, farnesyltransferase enzyme; GGT, geranygeranyl transferase; NLS, nuclear localization signal; PCBP1, poly (C)-binding protein 1; PPIase, peptidyl prolyl cis/trans isomerase; PRL, phosphatase of regenerating liver; PTP, protein tyrosine phosphatases; Src, sarcoma; STAT3, signal transducer activator of transcription 3; SUMO, small ubiquitin-like modifier; TGFβ, transforming growth factor β; TRP32, TRX-related protein 32; TrxR, TRX reductases; USP4, ubiquitin-specific protease 4; VEGF, vascular endothelial growth factor; UTR, untranslated region.

## Funding

EMBL–Marie Curie cofounded Interdisciplinary Postdoctoral (EIPOD) Fellowship to T.R.

## Competing Interests

The Authors declare that there are no competing interests associated with the manuscript.
